# Supplementary material for: Associations of Dynapenic Abdominal Obesity and Frailty Progression: Evidence from Two Nationwide Cohorts
Source: Nutrients. 2024 Feb 13;16(4):518. doi: 10.3390/nu16040518 (PMC10892768; doi:10.3390/nu16040518)
Supplement: Supplementary file 1 [file nutrients-16-00518-s001.zip › nutrients-2831778-supplementary.pdf]

Supplemental Materials

Supplemental Table S1. The detailed descriptions of FI in the CHARLS and ELSA.

Supplemental Table S2. Baseline characteristics of the study population stratified by dynapenia and abdominal obesity status.

Supplemental Table S3. Pairwise comparisons of baseline characteristics among dynapenia and abdominal obesity status in the CHARLS.

Supplemental Table S4. Pairwise comparisons of baseline characteristics among dynapenia and abdominal obesity status in the ELSA.

Supplemental Table S5. Comparisons of baseline characteristics between analytic samples and excluded samples with loss to follow up.

Supplemental Table S6. The detailed distributions of FI in the CHARLS and ELSA.

Supplemental Table S7. The deficits proportions across FI domains in the CHARLS and ELSA.

Supplemental Table S8. Associations of dynapenic abdominal obesity with FI progression among participants stratified by sex.

Supplemental Table S9. Associations of dynapenic abdominal obesity with FI progression among participants stratified by age group.

Supplemental Table S10. Numbers and percentages for the transitions of dynapenic abdominal obesity.

Supplemental Table S11. Intergroup comparisons for the FI progression among different dynapenic transitions.

Supplemental Table S12. Associations of dynapenic abdominal obesity with FI progression across FI domains.

Supplemental Table S13. Associations of dynapenia alone and abdominal obesity alone with FI progression.

Supplemental Table S14. Associations of dynapenia and obesity (BMI) with FI progression.

Supplemental Table S1. The detailed descriptions of FI in the CHARLS and ELSA

<table>
<tr><th rowspan="2">Domains</th><th colspan="2">Description of the items</th><th rowspan="2">Cut-off value</th></tr>
<tr><th>CHARLS</th><th>ELSA</th></tr>
<tr><td rowspan="13">Medical conditions</td><td colspan="2">Self-reported physician diagnosed hypertension</td><td rowspan="10">Yes = 1, No = 0</td></tr>
<tr><td colspan="2">Self-reported physician diagnosed diabetes</td></tr>
<tr><td colspan="2">Self-reported physician diagnosed heart disease</td></tr>
<tr><td colspan="2">Self-reported physician diagnosed stroke</td></tr>
<tr><td colspan="2">Self-reported physician diagnosed cancer</td></tr>
<tr><td colspan="2">Self-reported physician diagnosed arthritis</td></tr>
<tr><td colspan="2">Self-reported physician diagnosed chronic lung disease</td></tr>
<tr><td colspan="2">Self-reported physician diagnosed asthma</td></tr>
<tr><td colspan="2">Self-reported physician diagnosed any emotional, nervous, or psychiatric problems</td></tr>
<tr><td colspan="2">Self-reported physician diagnosed memory-related disease</td></tr>
<tr><td colspan="2">Self-reported vision problems/eyesight</td><td>Yes = 1, No = 0 in the CHARLS;
Poor or fair = 1, excellent, very good, o good = 0 in the ELSA</td></tr>
<tr><td colspan="2">Self-reported hearing problems/hearing</td><td>Yes = 1, No = 0 in the CHARLS;
Poor or fair = 1, excellent, very good, o good = 0 in the ELSA</td></tr>
<tr><td colspan="2">Self-reported general health status</td><td>Poor or fair = 1, excellent, very good, or good = 0</td></tr>
<tr><td rowspan="10">ADL/IADL disabilities</td><td colspan="2">Difficulty with dressing</td><td rowspan="10">Yes = 1, No = 0</td></tr>
<tr><td colspan="2">Difficulty with bathing or showering</td></tr>
<tr><td colspan="2">Difficulty with eating</td></tr>
<tr><td colspan="2">Difficulty with getting in and out of bed</td></tr>
<tr><td colspan="2">Difficulty with using the toilet</td></tr>
<tr><td colspan="2">Difficulty with managing money</td></tr>
<tr><td colspan="2">Difficulty with taking medications</td></tr>
<tr><td colspan="2">Difficulty with shopping for groceries</td></tr>
<tr><td colspan="2">Difficulty with preparing meals</td></tr>
<tr><td colspan="2">Difficulty with doing housework</td></tr>
<tr><td rowspan="7">Functional abilities</td><td colspan="2">Difficulty with walking 100 meters/yards</td><td rowspan="7">Yes = 1, No = 0</td></tr>
<tr><td colspan="2">Difficulty with getting up from a chair after sitting for long periods</td></tr>
<tr><td colspan="2">Difficulty with climbing several flights of stairs without resting</td></tr>
<tr><td colspan="2">Difficulty with lifting or carrying weights over 10 jins/pounds</td></tr>
<tr><td colspan="2">Difficulty with picking up a coin from the table</td></tr>
<tr><td colspan="2">Difficulty with stooping, kneeling, or crouching</td></tr>
<tr><td colspan="2">Difficulty with reaching arms above shoulder level</td></tr>
<tr><td>Depression</td><td>CESD-10</td><td>CESD-8</td><td>CESD-10 >10 =1, ≤10 =0 in the CHARLS;
CESD-8 ≥ 4 =1, <4 =0 in the ELSA</td></tr>
<tr><td>Cognition</td><td colspan="2">Sum of memory test score and orientation test score / 14</td><td>Continuous, ranging from 0 to 1</td></tr>
</table>

Notes: CHARLS, China Health and Retirement Longitudinal Study; ELSA, English Longitudinal Study of Aging; FI, frailty index; heart diseases included the angina, coronary heart disease, congestive heart failure, or other heart problems. Memory-related disease indicated Alzheimer's disease or dementia, organic brain senility, or other serious memory impairment. ADL, activities of daily living; IADL, instrumental activities of daily living; Depression was evaluated using Center for Epidemiologic Studies Depression Scale (CESD). In the CHARLS, CESD-10 was used, and the total score ranged from 0 to 30. In the ELSA, CESD-8 was used, and the total score ranges from 0 to 8. The higher score indicated more severe depressive symptoms. The memory score was the average number of words that were not recalled in the immediate and delayed word recall tasks. The memory score ranged from 0 to 10. The orientation test comprised four questions about the day of month, month, year, and the day of week. One point was given for each wrong answer, and the range is from 0 to 4.

Supplemental Table S2. Baseline characteristics of the study population stratified by dynapenia and abdominal obesity status

Variables	CHARLS (n=6937)				ELSA (n=3735)			
	ND/NAO	AO	D	D/AO	ND/NAO	AO	D	D/AO
Number, n (%)	3543 (51.1)	2630 (37.9)	499 (7.2)	265 (3.8)	1737(46.5)	1785(47.8)	97(2.6)	116(3.1)
Age, mean (SD), years	60.3 (7.0)	60.4 (6.9)	65.2 (8.3)	65.3 (8.5)	62.9 (7.6)	63.8 (7.9)	70.2 (9.4)	68.1 (8.8)
Female, n (%)	1455 (41.1)	1731 (65.8)	203 (40.7)	195 (73.6)	872 (50.2)	1077 (60.3)	64 (66.0)	88 (75.9)
Less than lower secondary, n (%)	3203 (90.4)	2381 (90.5)	484 (97.0)	253 (95.5)	383 (24.2)	593 (36.1)	43 (48.3)	61 (59.2)
Married or partnered, n (%)	3147 (88.8)	2341 (89.0)	403 (80.8)	203 (76.6)	1317 (75.8)	1287 (72.1)	50 (51.5)	60 (51.7)
Current smokers, n (%)	1437 (40.7)	531 (20.2)	203 (40.8)	49 (18.6)	225 (13.0)	223 (12.5)	15 (15.5)	16 (13.8)
Drinking at least once a month, n (%)	854 (25.8)	416 (16.5)	95 (20.3)	25 (9.6)	1335 (81.8)	1240 (74.9)	65 (74.7)	72 (67.3)
BMI, mean (SD), kg/m^2	21.5 (2.3)	26.0 (2.8)	20.8 (2.4)	26.0 (3.1)	24.8 (2.7)	31.1 (4.3)	24.2 (2.6)	30.9 (4.7)
waist circumferences, mean (SD), cm	78.0 (8.1)	94.7 (6.6)	76.9 (8.6)	95.4 (6.9)	86.6 (9.0)	103.9 (10.4)	84.8 (7.8)	101.8 (9.1)
Handgrip strength, mean (SD), kg	33.9 (8.9)	32.3 (9.2)	19.8 (5.2)	16.8 (4.3)	34.1 (10.5)	33.1 (11.1)	15.1 (4.8)	14.0 (4.4)
FI, median (IQR)	10.9 (5.4, 17.3)	13.8 (7.6, 20.6)	16.7 (8.5, 24.1)	20.4 (11.5, 32.9)	6.6 (3.6, 10.6)	10.4 (4.4, 19.5)	17.0 (10.5, 28.9)	28.9 (16.4, 38.5)
Frailty, n (%)	372 (10.5)	420 (16.0)	120 (24.0)	97 (36.6)	93 (5.4)	284 (15.9)	29 (29.9)	66 (56.9)

Notes: CHARLS, China Health and Retirement Longitudinal Study; ELSA, English Longitudinal Study of Aging; ND/NAO, non-dynapenia and non-abdominal obesity; AO, only abdominal obesity; D, only dynapenia; D/AO, dynapenic abdominal obesity; BMI, body mass index; FI, frailty index; Comparison of baseline characteristics among ND/NAO, AO, D, and D/AO are significant in the CHARLS (all $p<0.001$) and the ELSA except for current smokers ($p=0.189$)

Supplemental Table S3. Pairwise comparisons of baseline characteristics among dynapenia and abdominal obesity status in the CHARLS

Variables	ND/NAO	AO	D	D/AO	*P*1	*P*2	*P*3	*P*4	*P*5	*P*6
Age, mean (SD), years	60.3 (7.0)	60.4 (6.9)	65.2 (8.3)	65.3 (8.5)	0.640	<0.001	<0.001	<0.001	<0.001	0.810
Female, n (%)	1455 (41.1)	1731 (65.8)	203 (40.7)	195 (73.6)	<0.001	0.908	<0.001	<0.001	0.016	<0.001
Less than lower secondary, n (%)	3203 (90.4)	2381 (90.5)	484 (97.0)	253 (95.5)	0.792	<0.001	0.029	<0.001	0.029	0.424
Married or partnered, n (%)	3147 (88.8)	2341 (89.0)	403 (80.8)	203 (76.6)	0.848	<0.001	<0.001	<0.001	<0.001	0.251
Current smokers, n (%)	1437 (40.7)	531 (20.2)	203 (40.8)	49 (18.6)	<0.001	0.740	<0.001	<0.001	0.792	<0.001
Drinking at least once a month, n (%)	854 (25.8)	416 (16.5)	95 (20.3)	25 (9.6)	<0.001	0.026	<0.001	0.026	0.021	<0.001
BMI, mean (SD), kg/m^2	21.5 (2.3)	26.0 (2.8)	20.8 (2.4)	26.0 (3.1)	<0.001	<0.001	<0.001	<0.001	0.925	<0.001
waist circumferences, mean (SD), cm	78.0 (8.1)	94.7 (6.6)	76.9 (8.6)	95.4 (6.9)	<0.001	0.002	<0.001	<0.001	0.184	<0.001
Handgrip strength mean (SD), kg	33.9 (8.9)	32.3 (9.2)	19.8 (5.2)	16.8 (4.3)	<0.001	<0.001	<0.001	<0.001	<0.001	<0.001
FI, median (IQR)	10.9 (5.4, 17.3)	13.8 (7.6, 20.6)	16.7 (8.5, 24.1)	20.4 (11.5, 32.9)	<0.001	<0.001	<0.001	<0.001	<0.001	<0.001
Frailty, n (%)	372 (10.5)	420 (16.0)	120 (24.0)	97 (36.6)	<0.001	<0.001	<0.001	<0.001	<0.001	<0.001

Notes: CHARLS, China Health and Retirement Longitudinal Study; ND/NAO, non-dynapenia and non-abdominal obesity; AO, only abdominal obesity; D, only dynapenia; D/AO, dynapenic abdominal obesity; *P*1 was the *P* value for comparison between ND/NAO and AO; *P*2 was the *P* value for comparison between ND/NAO and D; *P*3 was the *P* value for comparison between ND/NAO and D/AO; *P*4 was the *P* value for comparison between AO and D; *P*5 was the *P* value for comparison between AO and D/AO; *P*6 was the *P* value for comparison between D and D/AO. All *P* values were adjusted by the false discovery rate (FDR) method.

Supplemental Table S4. Pairwise comparisons of baseline characteristics among dynapenia and abdominal obesity status in the ELSA

Variables	ND/NAO	AO	D	D/AO	*P*1	*P*2	*P*3	*P*4	*P*5	*P*6
Age, mean (SD), years	62.9 (7.6)	63.8 (7.9)	70.2 (9.4)	68.1 (8.8)	<0.001	<0.001	<0.001	<0.001	<0.001	0.053
Female, n (%)	872 (50.2)	1077 (60.3)	64 (66.0)	88 (75.9)	<0.001	0.005	<0.001	0.317	0.003	0.181
High school or above, n (%)	383 (24.2)	593 (36.1)	43 (48.3)	61 (59.2)	<0.001	<0.001	<0.001	0.032	<0.001	0.314
Married or partnered, n (%)	1317 (75.8)	1287 (72.1)	50 (51.5)	60 (51.7)	0.016	<0.001	<0.001	<0.001	<0.001	1.000
Current smokers, n (%)	225 (13.0)	223 (12.5)	15 (15.5)	16 (13.8)	—	—	—	—	—	—
Drink at least once a month, n (%)	1335 (81.8)	1240 (74.9)	65 (74.7)	72 (67.3)	<0.001	0.388	0.003	0.966	0.304	0.612
BMI, mean (SD), kg/m^2	24.8 (2.7)	31.1 (4.3)	24.2 (2.6)	30.9 (4.7)	<0.001	0.150	<0.001	<0.001	0.460	<0.001
waist circumferences, mean (SD), cm	86.6 (9.0)	103.9 (10.4)	84.8 (7.8)	101.8 (9.1)	<0.001	0.072	<0.001	<0.001	0.028	<0.001
Handgrip strength mean (SD), kg	34.1 (10.5)	33.1 (11.1)	15.1 (4.8)	14.0 (4.4)	0.006	<0.001	<0.001	<0.001	<0.001	0.482
FI, median (IQR)	6.6 (3.6, 10.6)	10.4 (4.4, 19.5)	17.0 (10.5, 28.9)	28.9 (16.4, 38.5)	<0.001	<0.001	<0.001	<0.001	<0.001	<0.001
Frailty, n (%)	93 (5.4)	284 (15.9)	29 (29.9)	66 (56.9)	<0.001	<0.001	<0.001	<0.001	<0.001	<0.001

Notes: ELSA, English Longitudinal Study of Aging; ND/NAO, non-dynapenia and non-abdominal obesity; AO, only abdominal obesity; D, only dynapenia; D/AO, dynapenic abdominal obesity; BMI, body mass index; FI, frailty index. ND/NAO, non-dynapenia/non-abdominal obesity; AO, only abdominal obesity; D, only dynapenia; D/AO, dynapenia/abdominal obesity; *P*1 was the *P* value for comparison between ND/NAO and AO; *P*2 was the *P* value for comparison between ND/NAO and D; *P*3 was the *P* value for comparison between ND/NAO and D/AO; *P*4 was the *P* value for comparison between AO and D; *P*5 was the *P* value for comparison between AO and D/AO; *P*6 was the *P* value for comparison between D and D/AO. All *P* values were adjusted by the false discovery rate (FDR) method.

Supplemental Table S5. Comparisons of baseline characteristics between analytic samples and excluded samples with loss to follow up

Variables	CHARLS		*P*	ELSA		*P*
	Analytic sample (n=6937)	Excluded sample (n=2759)		Analytic sample (n=3735)	Excluded sample (n=3492)	
Age, mean (SD), years	60.9 (7.3)	64.4 (9.5)	<0.001	63.7 (8.0)	68.9 (10.2)	<0.001
Female, n (%)	3584 (51.7)	1256 (45.5)	<0.001	2101 (56.3)	1823 (52.2)	0.001
Less than lower secondary, n (%)	6321 (91.1)	2470 (89.5)	<0.001	1080 (31.6)	1601 (50.6)	<0.001
Married or partnered, n (%)	6094 (87.8)	2182 (79.1)	<0.001	2714 (72.7)	2317 (66.4)	<0.001
Current smokers, n (%)	2220 (32.1)	887 (32.5)	<0.001	479 (12.8)	564 (16.2)	<0.001
Drinking at least once a month, n (%)	1390 (21.2)	529 (20.3)	0.631	2712 (77.9)	2140 (70.6)	<0.001
BMI, mean (SD), kg/m^2	23.3 (3.4)	23.1 (3.5)	0.009	28.0 (4.8)	27.8 (4.8)	0.141
waist circumferences, mean (SD), cm	85.0 (11.3)	84.9 (11.5)	0.971	95.3 (13.0)	96.3 (13.4)	0.001
Handgrip strength, mean (SD), kg	31.6 (9.8)	30.1 (10.1)	<0.001	32.5 (11.4)	29.8 (11.4)	<0.001
FI, median (IQR)	11.4 (7.5, 20.3)	14.2 (7.9, 23.8)	<0.001	7.5 (4.0, 16.5)	13.2 (6.8, 24.2)	<0.001
Frailty, n (%)	1009 (14.5)	651 (23.6)	<0.001	472 (12.6)	867 (24.8)	<0.001

Notes: CHARLS, China Health and Retirement Longitudinal Study; ELSA, English Longitudinal Study of Aging; BMI, body mass index; FI, frailty index.

Supplemental Table S6. The detailed distributions of FI in the CHARLS and ELSA

Variables	CHARLS (n=6937)				ELSA (n=3735)			
	ND/NAO	AO	D	D/AO	ND/NAO	AO	D	D/AO
Medical conditions								
Hypertension, n (%)	633 (17.9)	991 (37.7)	106 (21.2)	112 (42.3)	474 (27.3)	773 (43.3)	30 (30.9)	62 (53.4)
Diabetes, n (%)	117 (3.3)	253 (9.6)	26 (5.2)	25 (9.4)	61 (3.5)	143 (8.0)	2 (2.1)	15 (12.9)
Heart disease, n (%)	320 (9.0)	416 (15.8)	49 (9.8)	41 (15.5)	204 (11.7)	219 (12.3)	12 (12.4)	21 (18.1)
Stroke, n (%)	68 (1.9)	69 (2.6)	12 (2.4)	12 (4.5)	35 (2.0)	48 (2.7)	6 (6.2)	5 (4.3)
Cancer, n (%)	24 (0.7)	21 (0.8)	3 (0.6)	3 (1.1)	98 (5.6)	122 (6.8)	8 (8.2)	5 (4.3)
Arthritis, n (%)	1187 (33.5)	958 (36.4)	202 (40.5)	112 (42.3)	397 (22.9)	647 (36.2)	58 (59.8)	80 (69.0)
Chronic lung disease, n (%)	367 (10.4)	214 (8.1)	59 (11.8)	33 (12.5)	61 (3.5)	88 (4.9)	4 (4.1)	7 (6.0)
Asthma, n (%)	159 (4.5)	120 (4.6)	36 (7.2)	14 (5.3)	172 (9.9)	250 (14.0)	10 (10.3)	16 (13.8)
Psychiatric problems, n (%)	38 (1.1)	35 (1.3)	6 (1.2)	2 (0.8)	128 (7.4)	163 (9.1)	7 (7.2)	7 (6.0)
Memory-related disease, n (%)	33 (0.9)	33 (1.3)	15 (3.0)	12 (4.5)	3 (0.2)	4 (0.2)	0 (0.0)	0 (0.0)
Vision problems, n (%)	233 (6.6)	135 (5.1)	33 (6.6)	18 (6.8)	121 (7.0)	194 (10.9)	17 (17.5)	23 (19.8)
Hearing problems, n (%)	315 (8.9)	213 (8.1)	51 (10.2)	32 (12.1)	278 (16.0)	344 (19.3)	24 (24.7)	28 (24.1)
Health status, n (%)	2734 (77.2)	2033 (77.3)	424 (85.0)	219 (82.6)	219 (12.6)	406 (22.7)	31 (32.0)	64 (55.2)
ADL/IADL disabilities								
Dressing, n (%)	113 (3.2)	85 (3.2)	40 (8.0)	26 (9.8)	76 (4.4)	226 (12.7)	20 (20.6)	46 (39.7)
Bathing, n (%)	117 (3.3)	113 (4.3)	44 (8.8)	41 (15.5)	54 (3.1)	146 (8.2)	20 (20.6)	36 (31.0)
Eating, n (%)	37 (1.0)	37 (1.4)	23 (4.6)	18 (6.8)	10 (0.6)	13 (0.7)	9 (9.3)	7 (6.0)
Getting in and out of bed, n (%)	111 (3.1)	106 (4.0)	43 (8.6)	32 (12.1)	31 (1.8)	92 (5.2)	11 (11.3)	17 (14.7)
Toilet using, n (%)	297 (8.4)	333 (12.7)	93 (18.6)	74 (27.9)	19 (1.1)	47 (2.6)	6 (6.2)	9 (7.8)
Managing money, n (%)	385 (10.9)	301 (11.4)	92 (18.4)	61 (23.0)	15 (0.9)	16 (0.9)	3 (3.1)	8 (6.9)
Taking medications, n (%)	195 (5.5)	136 (5.2)	46 (9.2)	29 (10.9)	11 (0.6)	15 (0.8)	3 (3.1)	2 (1.7)
Shopping, n (%)	214 (6.0)	171 (6.5)	59 (11.8)	50 (18.9)	40 (2.3)	82 (4.6)	19 (19.6)	32 (27.6)
Preparing meals, n (%)	184 (5.2)	147 (5.6)	61 (12.2)	53 (20.0)	22 (1.3)	29 (1.6)	9 (9.3)	10 (8.6)
Doing housework, n (%)	176 (5.0)	195 (7.4)	62 (12.4)	53 (20.0)	93 (5.4)	236 (13.2)	27 (27.8)	51 (44.0)

Functional abilities								
Walking 100 meters/yards, n (%)	40 (1.1)	42 (1.6)	9 (1.8)	10 (3.8)	58 (3.3)	144 (8.1)	20 (20.6)	34 (29.3)
Getting up from a chair after sitting for long periods, n (%)	1209 (34.1)	1215 (46.2)	256 (51.3)	173 (65.3)	304 (17.5)	690 (38.7)	48 (49.5)	82 (70.7)
Climbing several flights of stairs without resting, n (%)	816 (23.0)	827 (31.4)	196 (39.3)	139 (52.5)	232 (13.4)	498 (27.9)	38 (39.2)	66 (56.9)
Lifting or carrying weights over 10 jins/pounds, n (%)	893 (25.2)	906 (34.4)	197 (39.5)	139 (52.5)	345 (19.9)	722 (40.4)	51 (52.6)	89 (76.7)
Picking up a coin from the table, n (%)	275 (7.8)	242 (9.2)	83 (16.6)	49 (18.5)	107 (6.2)	156 (8.7)	22 (22.7)	34 (29.3)
Stooping, kneeling, or crouching, n (%)	257 (7.3)	248 (9.4)	94 (18.8)	66 (24.9)	172 (9.9)	363 (20.3)	53 (54.6)	76 (65.5)
Reaching arms above shoulder level, n (%)	83 (2.3)	64 (2.4)	40 (8.0)	16 (6.0)	35 (2.0)	51 (2.9)	18 (18.6)	33 (28.4)
Depression, n (%)	1268 (35.8)	888 (33.8)	251 (50.3)	133 (50.2)	170 (9.8)	237 (13.3)	25 (25.8)	25 (21.6)
Cognition score, mean (SD)	0.5 (0.1)	0.5 (0.1)	0.6 (0.1)	0.6 (0.1)	0.3 (0.1)	0.3 (0.1)	0.4 (0.1)	0.4 (0.1)

Notes: CHARLS, China Health and Retirement Longitudinal Study; ELSA, English Longitudinal Study of Aging; ND/NAO, non-dynapenia and non-abdominal obesity; AO, only abdominal obesity; D, only dynapenia; D/AO, dynapenic abdominal obesity; heart diseases consisted of the angina, coronary heart disease, congestive heart failure, and other problems. Memory-related disease included Alzheimer’s disease, dementia, and other memory impairment. ADL, activities of daily living; IADL, instrumental activities of daily living; Depression was evaluated using Center for Epidemiologic Studies Depression Scale (CESD). CESD-10 (ranging from 0 to 30) and CESD-8 (ranging from 0 to 8) were used in the CHARLS and ELSA, respectively. The higher score indicated more severe depressive symptoms. Cognition score was calculated through the memory and orientation test. The memory test was conducted by calculating the average number of words which were not recalled in the immediate and delayed word recall and the range was from 0 to 10. The orientation test comprised four questions about the day of the week, the month, the date of the month, and the year and the range was from 0 to 4.

Supplemental Table S7. The deficits proportions across FI domains in the CHARLS and ELSA

Deficits	CHARLS (n=6937)				ELSA (n=3735)			
	ND/NAO	AO	D	D/AO	ND/NAO	AO	D	D/AO
Medical conditions, n (%)								
0	460 (13.0)	297 (11.3)	41 (8.2)	18 (6.8)	553 (31.8)	347 (19.4)	10 (10.3)	6 (5.2)
1	1171 (33.1)	674 (25.6)	146 (29.3)	55 (20.8)	563 (32.4)	495 (27.7)	27 (27.8)	16 (13.8)
2	1125 (31.8)	737 (28.0)	157 (31.5)	76 (28.7)	346 (19.9)	394 (22.1)	26 (26.8)	30 (25.9)
3	491 (13.9)	536 (20.4)	97 (19.4)	65 (24.5)	157 (9.0)	256 (14.3)	14 (14.4)	26 (22.4)
4	193 (5.4)	255 (9.7)	37 (7.4)	32 (12.1)	78 (4.5)	169 (9.5)	13 (13.4)	20 (17.2)
≥5	103 (2.8)	131 (5.0)	21 (4.2)	19 (7.1)	40 (2.4)	124 (6.9)	7 (7.2)	18 (15.5)
ADL/IADL disabilities, n (%)								
0	2702 (76.3)	1906 (72.5)	309 (61.9)	138 (52.1)	1557 (89.6)	1369 (76.7)	57 (58.8)	42 (36.2)
1	435 (12.3)	354 (13.5)	73 (14.6)	39 (14.7)	101 (5.8)	198 (11.1)	14 (14.4)	21 (18.1)
2	170 (4.8)	172 (6.5)	39 (7.8)	24 (9.1)	34 (2.0)	94 (5.3)	6 (6.2)	19 (16.4)
3	97 (2.7)	73 (2.8)	17 (3.4)	13 (4.9)	15 (0.9)	48 (2.7)	7 (7.2)	6 (5.2)
4	52 (1.5)	36 (1.4)	22 (4.4)	12 (4.5)	12 (0.7)	39 (2.2)	4 (4.1)	15 (12.9)
≥5	87 (2.4)	89 (3.4)	39 (7.8)	39 (14.9)	18 (1.2)	37 (2.0)	9 (9.3)	13 (11.2)
Functional abilities, n (%)								
0	1791 (50.6)	1050 (39.9)	171 (34.3)	62 (23.4)	1109 (63.8)	730 (40.9)	20 (20.6)	9 (7.8)
1	748 (21.1)	560 (21.3)	89 (17.8)	48 (18.1)	311 (17.9)	354 (19.8)	17 (17.5)	11 (9.5)
2	477 (13.5)	410 (15.6)	76 (15.2)	32 (12.1)	151 (8.7)	244 (13.7)	18 (18.6)	20 (17.2)
3	325 (9.2)	373 (14.2)	70 (14.0)	53 (20.0)	81 (4.7)	214 (12.0)	11 (11.3)	13 (11.2)
4	129 (3.6)	160 (6.1)	54 (10.8)	37 (14.0)	44 (2.5)	122 (6.8)	9 (9.3)	18 (15.5)
≥5	73 (2.1)	77 (3.0)	39 (7.8)	33 (12.4)	41 (2.4)	121 (6.7)	22 (22.7)	45 (38.8)

Notes: CHARLS, China Health and Retirement Longitudinal Study; ELSA, English Longitudinal Study of Aging; ND/NAO, non-dynapenia and non-abdominal obesity; AO, only abdominal obesity; D, only dynapenia; D/AO, dynapenic abdominal obesity; ADL, activities of daily living; IADL, instrumental activities of daily living.

Supplemental Table S8. Associations of dynapenic abdominal obesity with FI progression stratified by sex

Sex	Variables	CHARLS			ELSA		
		β	95% CI	*p*	*β*	95% CI	*p*
Male	ND/NAO×time	Reference	-	-	Reference	-	-
	AO×time	0.170	0.039 to 0.312	0.012	0.108	0.005 to 0.212	0.041
	D×time	0.476	0.109 to 0.550	0.003	0.422	0.057 to 0.788	0.024
	D/AO×time	0.612	-0.047 to 0.786	0.083	0.348	-0.060 to 0.756	0.095
Female	ND/NAO×time	Reference	-	-	Reference	-	-
	AO×time	0.159	0.022 to 0.295	0.023	0.224	0.125 to 0.323	<0.001
	D×time	0.431	0.135 to 0.726	0.004	0.529	0.242 to 0.817	<0.001
	D/AO×time	0.305	0.015 to 0.595	0.039	0.269	0.023 to 0.514	0.032

Notes: FI, frailty index; CHARLS, China Health and Retirement Longitudinal Study; ELSA, English Longitudinal Study of Aging; ND/NAO, non-dynapenia and non-abdominal obesity; AO, only abdominal obesity; D, only dynapenia; D/AO, dynapenic abdominal obesity; The *β* and *p* were adjusted for age, sex, education level, marital tatus, smoking status, drinking status, and body mass index (BMI). Results were additionally adjusted for non-response household and individual weights (CHARLS) and person-level nurse interview weight (ELSA).

Supplemental Table S9. Associations of dynapenic abdominal obesity with FI progression stratified by age group

Age group	Variables	CHARLS			ELSA		
		β	95% CI	p	β	95% CI	p
50-60	ND/NAO×time	Reference	-	-	Reference	-	-
	AO×time	0.229	0.115 to 0.344	<0.001	0.132	0.046 to 0.218	0.003
	D×time	0.430	0.141 to 0.719	0.004	-0.666	-1.074 to -0.259	0.001
	D/AO×time	0.123	-0.254 to 0.501	0.521	-0.234	-0.592 to 0.123	0.199
60-70	ND/NAO×time	Reference	-	-	Reference	-	-
	AO×time	0.263	0.104 to 0.422	0.001	0.108	0.003 to 0.213	0.045
	D×time	0.387	0.066 to 0.708	0.018	0.189	-0.164 to 0.541	0.294
	D/AO×time	0.393	-0.018 to 0.804	0.061	-0.116	-0.423 to 0.190	0.458
70+	ND/NAO×time	Reference	-	-	Reference	-	-
	AO×time	-0.076	-0.420 to 0.268	0.664	0.197	0.001 to 0.393	0.049
	D×time	-0.122	-0.599 to 0.355	0.616	0.680	0.258 to 1.103	0.002
	D/AO×time	0.131	-0.459 to 0.721	0.663	0.447	0.030 to 0.864	0.036

Notes: FI, frailty index; CHARLS, China Health and Retirement Longitudinal Study; ELSA, English Longitudinal Study of Aging; ND/NAO, non-dynapenia and non-abdominal obesity; AO, only abdominal obesity; D, only dynapenia; D/AO, dynapenic abdominal obesity; The β and p were adjusted for age, sex, education level, marital tatus, smoking status, drinking status, and body mass index (BMI). Results were additionally adjusted for non-response household and individual weights (CHARLS) and person-level nurse interview weight (ELSA).

Supplemental Table S10. Numbers and percentages for the transitions of dynapenic abdominal obesity status

Dynapenic abdominal obesity status at baseline	Dynapenic abdominal obesity status at the second resurvey, n (%)			
	ND/NAO	AO	D	D/AO
CHARLS				
ND/NAO	1968(70.1)	560(19.9)	228(8.1)	52(1.9)
AO	272(13.2)	1631(79.4)	22(1.2)	128(6.2)
D	166(45.4)	58(15.8)	120(32.8)	22(6.0)
D/AO	12(6.3)	121(63.4)	8(4.2)	50(26.1)
ELSA				
ND/NAO	1205(75.0)	311(19.4)	65(4.0)	26(1.6)
AO	159(9.7)	1357(83.7)	11(0.7)	95(5.9)
D	26(35.6)	10(13.7)	27(37.0)	10(13.7)
D/AO	7(8.0)	30(34.0)	7(8.0)	44(50.0)

Notes: The second resurvey is at wave 3 for the CHARLS and wave 4 for the ELSA with four-year interval; Row percentages were reported; CHARLS, China Health and Retirement Longitudinal Study; ELSA, English Longitudinal Study of Aging; ND/NAO, non-dynapenia and non-abdominal obesity; AO, only abdominal obesity; D, only dynapenia; D/AO, dynapenic abdominal obesity.

Supplemental Table S11**.** Intergroup comparisons for the FI progression among different dynapenic transitions

Dynapenic transitions	CHARLS		ELSA	
	β (95% CI)	*p*	*β* (95% CI)	*p*
Stable ND/NAO×Time	Reference		Reference	
ND/NAO to D×Time	0.295(0.059 to 0.531)	0.014	0.262(0.059 to 0.531)	0.014
Stable D×Time	Reference		Reference	
D to ND/NAO×Time	-0.289(-0.811 to 0.234)	0.280	0.118(-0.582 to 0.819)	0.742
Stable AO×Time	Reference		Reference	
AO to D/AO×Time	0.150(-0.171 to 0.471)	0.360	0.522(0.278 to 0.765)	<0.001
Stable D/AO×Time	Reference		Reference	
D/AO to AO×Time	-0.436(-1.177 to 0.306)	0.251	-0.502(-1.164 to 0.159)	0.141

Notes: FI, frailty index; CHARLS, China Health and Retirement Longitudinal Study; ELSA, English Longitudinal Study of Aging; ND/NAO, non-dynapenia and non-abdominal obesity; AO, only abdominal obesity; D, only dynapenia; D/AO, dynapenic abdominal obesity; The *β* and *p* were adjusted for age, sex, education level, marital status, smoking status, drinking status, and body mass index (BMI). Results were additionally adjusted for non-response household and individual weights (CHARLS) and person-level nurse interview weight (ELSA).

Supplemental Table S12. Associations of dynapenic abdominal obesity with FI progression across FI domains

Domains	Variables	CHARLS			ELSA		
		β	95% CI	*p*	*β*	95% CI	*p*
Medical conditions	ND/NAO×time	Reference	-	-	Reference	-	-
	AO×time	0.030	0.020 to 0.041	<0.001	0.024	0.015 to 0.032	<0.001
	D×time	-0.003	-0.021 to 0.016	0.783	0.054	0.023 to 0.081	<0.001
	D/AO×time	0.036	0.012 to 0.060	0.003	0.025	-0.001 to 0.050	0.053
ADL/IADL disabilities	ND/NAO×time	Reference	-	-	Reference	-	-
	AO×time	0.026	0.010 to 0.042	0.001	0.011	0.002 to 0.022	0.022
	D×time	0.099	0.069 to 0.129	<0.001	0.053	0.021 to 0.086	0.001
	D/AO×time	0.038	-0.001 to 0.077	0.051	0.044	0.014 to 0.074	0.004
Functional abilities	ND/NAO×time	Reference	-	-	Reference	-	-
	AO×time	0.008	-0.006 to 0.021	0.250	0.019	0.009 to 0.029	<0.001
	D×time	0.041	0.015 to 0.066	0.002	0.043	0.011 to 0.076	0.009
	D/AO×time	0.057	0.024 to 0.090	<0.001	0.018	-0.012 to 0.048	0.251
Depression	ND/NAO×time	Reference					
	AO×time	0.004	0.000 to 0.008	0.065	0.00002	-0.002 to 0.002	0.985
	D×time	-0.013	-0.021 to -0.005	0.002	0.001	-0.009 to 0.006	0.743
	D/AO×time	-0.006	-0.016 to 0.004	0.233	0.0002	-0.007 to 0.007	0.948
Cognition	ND/NAO×time						
	AO×time	0.001	-0.001 to 0.002	0.096	-0.001	-0.001 to 0.001	0.979
	D×time	-0.002	-0.004 to 0.001	0.134	0.004	0.001 to 0.006	0.013
	D/AO×time	-0.001	-0.004 to 0.003	0.906	-0.001	-0.003 to 0.002	0.857

Notes: FI, frailty index; CHARLS, China Health and Retirement Longitudinal Study; ELSA, English Longitudinal Study of Aging; ND/NAO, non-dynapenia and non-abdominal obesity; AO, only abdominal obesity; D, only dynapenia; D/AO, dynapenic abdominal obesity; ADL, activities of daily living; IADL, instrumental activities of daily living. The *β* and *p* were adjusted for age, sex, education level, marital status, smoking status, drinking status, and waist circumferences. Results were additionally adjusted for non-response household and individual weights (CHARLS) and person-level nurse interview weight (ELSA).

Supplemental Table S13. Associations of dynapenia alone and abdominal obesity alone with FI progression

Variables	CHARLS			ELSA		
	β	95% CI	*p*	*β*	95% CI	*p*
Time, years	0.781	0.720 to 0.842	<0.001	0.455	0.405 to 0.504	<0.001
ND/NAO	Reference	-	-	Reference	-	-
AO alone	0.088	-0.574 to 0.751	0.794	1.429	0.515 to 2.343	0.009
D alone	4.276	3.478 to 5.074	<0.001	11.382	9.852 to 12.912	<0.001
ND/NAO×time	Reference	-	-	Reference	-	-
AO×time	0.194	0.104 to 0.283	<0.001	0.149	0.080 to 0.218	<0.001
D×time	0.293	0.151 to 0.436	<0.001	0.276	0.124 to 0.429	<0.001

Notes: FI, frailty index; CHARLS, China Health and Retirement Longitudinal Study; ELSA, English Longitudinal Study of Aging; AO: abdominal obesity alone; D: dynapenia alone; The *β* and *p* were adjusted for age, sex, education level, marital status, smoking status, drinking status, and body mass index (BMI). Results were additionally adjusted for non-response household and individual weights (CHARLS) and person-level nurse interview weight (ELSA).

Supplemental Table S14. Associations of dynapenia and obesity (BMI) with FI progression

Variables	CHARLS			ELSA		
	β	95% CI	*p*	*β*	95% CI	*p*
Time, years	0.822	0.766 to 0.879	<0.001	0.483	0.441 to 0.525	<0.001
ND/NO	Reference	-	-	Reference	-	-
O	1.137	0.492 to 1.783	<0.001	0.425	-0.624 to 1.474	0.427
D	3.608	2.686 to 4.531	<0.001	10.657	8.837 to 12.477	<0.001
D/O	7.176	5.623 to 8.728	<0.001	13.213	10.433 to 15.992	<0.001
ND/NO×time	Reference	-	-	Reference	-	-
O×time	0.138	0.038 to 0.239	0.007	0.166	0.087 to 0.245	<0.001
D×time	0.352	0.187 to 0.517	<0.001	0.392	0.209 to 0.576	<0.001
D/O×time	0.235	-0.042 to 0.511	0.097	0.186	-0.085 to 0.457	0.179

Notes: FI, frailty index; CHARLS, China Health and Retirement Longitudinal Study; ELSA, English Longitudinal Study of Aging; ND/NO, non-dynapenia/non-obesity; O, only obesity; D, only dynapenia; D/O, dynapenia/obesity; The *β* and *p* were adjusted for age, sex, education level, marital status, smoking status, drinking status, and waist circumferences. Obesity status was measured by BMI based on country-specific levels. Results were additionally adjusted for non-response household and individual weights (CHARLS) and person-level nurse interview weight (ELSA).
